# Pain in the Developing Brain: Early Life Factors Alter Nociception and Neurobiological Function in Adolescent Rats

**DOI:** 10.1093/texcom/tgab014

**Published:** 2021-02-24

**Authors:** Sabrina Salberg, Glenn R Yamakawa, Yannick Griep, Jesse Bain, Jaimie K Beveridge, Mujun Sun, Stuart J McDonald, Sandy R Shultz, Rhys D Brady, David K Wright, Melanie Noel, Richelle Mychasiuk

**Affiliations:** 1 Department of Neuroscience, Monash University, Melbourne 3004, Australia; 2 Behavioural Science Institute, Radboud University, Nijmegen 6525 GD, the Netherlands; 3 Division of Epidemiology, Stress Research Institute, Stockholm University, Stockholm 114 19, Sweden; 4 Department of Psychology, University of Calgary, Calgary T2N 1N4, Canada; 5 Department of Physiology, Anatomy and Microbiology, La Trobe University, Melbourne 3086, Australia

**Keywords:** corticospinal tract, early life stress, high-fat high-sugar diet, MRI, plantar incision surgery

## Abstract

Although adverse early experiences prime individuals to be at increased risk for chronic pain, little research has examined the trauma–pain relationship in early life or the underlying mechanisms that drive pathology over time. Given that early experiences can potentiate the nociceptive response, this study aimed to examine the effects of a high-fat, high-sugar (HFHS) diet and early life stress (maternal separation [MS]) on pain outcomes in male and female adolescent rats. Half of the rats also underwent a plantar-incision surgery to investigate how the pain system responded to a mildly painful stimuli in adolescence. Compared with controls, animals that were on the HFHS diet, experienced MS, or had exposure to both, exhibited increased anxiety-like behavior and altered thermal and mechanical nociception at baseline and following the surgery. Advanced magnetic resonance imaging demonstrated that the HFHS diet and MS altered the maturation of the brain, leading to changes in brain volume and diffusivity within the anterior cingulate, amygdala, corpus callosum, nucleus accumbens, and thalamus, while also modifying the integrity of the corticospinal tracts. The effects of MS and HFHS diet were often cumulative, producing exacerbated pain sensitivity and increased neurobiological change. As early experiences are modifiable, understanding their role in pain may provide targets for early intervention/prevention.

## Introduction

Chronic pain is one of the most prevalent and expensive health conditions affecting adolescents (King et al. 2011). Although acute pain is necessary for survival, chronic pain is often dysfunctional, highly debilitating, and generally emerges in late childhood or early adolescence (Palermo 2000; Melzack 2001; King et al. 2011). Adolescence is a critical developmental period that results in maturational changes in both the body and brain (Spear 2013). These include changes in hormone levels, the development of secondary sex characteristics, as well as increased myelination and synaptic pruning that remodel the neurocircuitry of the brain (Giedd 2008; Steinberg et al. 2008; Blakemore et al. 2010). Given the significant development that occurs during this period, environmental factors have the potential to substantially modify long-term trajectories. This can occur via programming mechanisms where, during critical periods of plasticity, organisms are primed to adapt to their environment in an effort to increase their propensity for survival (Welberg and Seckl 2001). However, if circumstances early in life do not match with those in their later environment, these adaptations can be maladaptive and prime systems to inappropriately respond to stimuli (Welberg and Seckl 2001).

Since pediatric pain conditions most often emerge in the adolescent period (King et al. 2011), early life factors pose promise as both mechanisms for impairment and targets for prevention and intervention. Two early life factors that may affect pain outcomes include: 1) consumption of a high-fat, high-sugar (HFHS) diet and 2) adverse childhood experiences (ACEs). Common western diets consisting of high levels of fats and sugars can cause chronic low-grade inflammation and lead to persistent microglia activation (Calder et al. 2011; Buckman et al. 2014; Kopp 2019). When microglia are persistently activated, they overproduce proinflammatory cytokines (Hains et al. 2010; Edlow et al. 2019), which may inappropriately activate nociceptive sensory neurons and potentiate pain responsivity (Hains and Waxman 2006; Zhang and An 2007). Similarly, ACEs, such as abuse, neglect, or household dysfunction (Dennis et al. 2019), have been shown to promote the basal proinflammatory states within the brain (Diz-Chaves et al. 2013), modify the stress response, hypothalamic–pituitary–adrenal (HPA) axis function, and cortisol and glucocorticoid receptor levels (Kalinichev et al. 2002; Aisa et al. 2007). Given this, these early experiences have the potential to modify the development of neurological systems responsible for nociception and have been shown to structurally and functionally alter the brain regions that are important for these processes (Cohen et al. 2006; Spinelli et al. 2009; Duque et al. 2012; Baker et al. 2013; Hanson et al. 2013; Howell et al. 2013; Lou et al. 2014; Saleh et al. 2017; Croll et al. 2018).

Therefore, the aim of this study was to examine the effects of a HFHS diet and an ACE on pain outcomes in adolescence. Outcomes were assessed at baseline and following an acutely painful stimulus in adolescent rats. These assessment points were chosen to evaluate the underlying changes in nociception and investigate how the system responded to a mildly painful stimuli later in life. We employed a combination of behavioral measures (thermal and mechanical nociception as well as anxiety-like behavior) and advanced magnetic resonance imaging (MRI) techniques (volumetric, diffusion tensor imaging, and tractography) to generate a comprehensive understanding of the neurodevelopmental changes that occur in the pain system in response to early life factors.

## Methods

### Animals, Diets, and Maternal Separation

All experiments were carried out in accordance with the Precinct Animal Centre (PAC) Animal Care and were approved by the Alfred Medical Research and Education Precinct (AMREP) Animal Ethics Committee.

Sprague-Dawley dams (8F) and sires (8M) were obtained from the Monash Animal Research Platform. Pups (27 female, 27 male) were in-house bred from the aforementioned dams and sires in the PAC. Animals were maintained on a 12:12-h light:dark cycle (lights on 0600) in a temperature-controlled (21 °C) animal facility (PAC).

Prior to mating, dams were assigned to either a standard diet (4F) (specialty feeds; 20% protein, 4.8% fat, 4.8% crude fiber, 0.36% salt, 14 MJ/kg digestible energy) or HFHS diet (4F) ([specialty feeds; 19.40% protein, 60.00% fat, 4.70% crude fiber, 4.70% axial diffusivity (AD) fiber, 24 MJ/kg digestible energy; 81% total calculated digestible energy from lipids, 13% total calculated digestible energy from protein) + (20% sugar water]). All sires were maintained on the standard diet and were mated with the dams for 5 days. Dams were maintained on their respective diets throughout gestation and until they were euthanized.

Pups were randomly assigned to either a control or maternal separation (MS) paradigm (Salberg et al. 2020). Those in the MS group were removed from their mother and placed together in an empty, half-heated cage for 4 h/day (1000–1400) for 12 days (from postnatal days [p] 2–13). The control pups were left undisturbed with their mothers.

**Figure 1:**
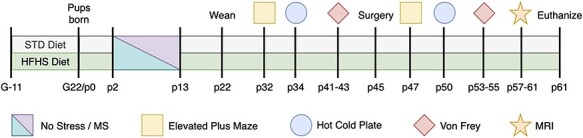
Experimental timeline illustrating order of procedures, with G indicating gestational day for dams and p indicating postnatal day for pups. STD diet, standard diet; HFHS Diet, High-Fat High-Sugar Diet; MS, Maternal Separation.

At weaning (p22), pups were maintained on the diet which their mother was assigned to, following which a baseline battery of behavior tests was performed on all pups. The pups were further randomized to receive either a sham injury or plantar incision surgery (p45), which was followed by a second postsurgical battery of behavior tests. No more than two pups from each litter were randomized into any given group. See Figure 1 for the illustrative timeline of experimental manipulations for all animals.

### Plantar Incision Surgery

The plantar incision surgery was performed on p45 as described by Brennan et al. (1996). Briefly, animals were anesthetized with 5% isoflurane at 1 L/min of O_2_. Once induced, animals were transferred to a nose-cone with 2% isoflurane at 1 L/min of O_2_ for the duration of the procedure. The left hind paw was sterilized with chlorhexidine and alcohol, following which a ~1 cm longitudinal incision was made in the skin from the mid-heel to the first foot pad. A second longitudinal incision was made in the underlying plantaris muscle. The incision was closed with two simple interrupted sutures. A subcutaneous injection of paracetamol (200 mg/kg) was given for postoperative analgesia. Isoflurane was turned off, and animals were allowed to recover in a clean, empty, and half-heated cage. The animal was returned to its’ home-cage once conscious and recovered. For the sham injury, animals were anesthetized with 5% isoflurane at 1 L/min of O_2_ and then allowed to recover in a clean, empty, half-heated cage, and then returned to their home-cage.

### Elevated Plus Maze

The elevated plus maze (EPM) task was run as a measure for anxiety-like behavior (Walf and Frye 2007). The maze consisted of two open arms (no vertical walls) and two closed arms (enclosed in vertical walls). All arms intersect at a center platform, to form the plus shape. The maze was constructed of black Plexiglas raised 51 cm above the ground, with each arm measuring 51 × 11 cm and with a center of 11 × 11 cm. A single, 5-min trial was run at each time (baseline and postsurgical), which began by placing the rat in the center of the maze, facing a closed arm, and allowing it to explore. The trial was recorded using an overhead camera and TopScan tracking software. This software tracked the nose of the animal throughout the trial and calculated time spent in the open arms, closed arms, and center. Higher anxiety is indicated by a less amount of time spent in the openarms.

### Hot Cold Plate

The hot cold plate was used as a measure of thermal nociceptive responsivity (Woolfe and MacDonald 1944; Bennett and Xie 1988; Le Bars et al. 2001). The apparatus consisted of a circular, temperature-controlled plate, enclosed with a transparent cylinder. At each time, the animal was habituated to the apparatus for 2 consecutive days before being tested the following day. On habituation days, the animal was placed on the room-temperature plate for 2 min, then returned to its’ home-cage. On testing day, the plate was first set to hot (52 °C), the animal was placed inside, and latency to react was recorded. The animal was immediately removed from the plate and returned to its home-cage once it reacted. The animal was then left undisturbed for >1 h, following which the same process was repeated with the plate set to cold (2 °C). Greater latency to react was indicative of increased thermal nociceptive thresholds.

### von Frey

The von Frey was run as a measure of mechanical nociceptive responsivity (Chaplan et al. 1994; Le Bars et al. 2001). The apparatus consisted of a grated stage (L72 × W36 × H37 cm), with a grid of 0.6 × 0.6 cm squares attached to the top of the stage. Enclosed boxes were place on top of the grid to contain the rat. At each time, the animal was habituated over 2 consecutive days by being placed inside the box and left undisturbed for 20 min, then being returned to its’ home-cage. On the testing day, the animal was again habituated to the box for 20 min, following which a series of increasingly large filaments were applied to its’ hind paws. Each filament was applied 5× to each hind paw and the number of reactions (withdrawal of hind paw) were recorded. Increasing sizes of filaments were used until the number of reactions for each paw was 5/5; this filament size was recorded. Larger filament size indicated a greater mechanical nociceptive threshold.

### Magnetic Resonance Imaging

Before the scan, the animal was anesthetized in an induction chamber with 5% isoflurane at 1 L/min of O_2_. After induction, the animal was transferred to a nose-cone with 2–3% isoflurane (depending on the size of the animal) at 1 L/min of O_2_ to maintain anesthesia for the duration of the scan. Respiration rate and temperature of the animal were monitored throughout the scan, with probes being positioned under the chest and in the rectum, respectively. Anesthesia was adjusted as needed in response to changes in respiration as was the warm water bath that ran underneath the animal in response to body temperature changes. Eye gel was applied to prevent the eyes from drying out, and ear bars were positioned to reduce movement during the scan. Once the animal was properly secured, it was positioned inside a 9.4T Bruker MRI for imaging with actively decoupled volume transmit and cryogenically cooled surface array coils. A T2-weighted image was acquired in the coronal plane using a rapid acquisition with relaxation enhancement (RARE) sequence for volumetric analysis. Twenty-four slices that were 0.8 mm thick were acquired with the following imaging parameters: repetition time = 4500 ms; effective echo time (time echo [TE]) = 45 ms; RARE factor = 8; field of view (FOV) = 28.8 × 28.8 mm^2^; and resolution = 75 × 75 um^2^. Diffusion-weighted images (DWI) were acquired over 81 directions with a *b*-value of 3000 s/mm^2^ using a 2D echo-planar sequence. Imaging parameters included: TR = 4000 ms, TE = 29 ms, and FOV = 28.8 × 28.8 mm^2^.

For the analysis, T2-weighted images were manually segmented using ITKsnap and volumes were calculated using FSL. Regions of interest (ROIs) included the anterior cingulate cortex (ACC), amygdala, corpus callosum (CC), nucleus accumbens (NAc), and thalamus. DWI processing was performed using MRtrix3 as described previously (Wright et al. 2019). Mean diffusion tensor metrics, including fractional anisotropy (FA), apparent diffusion coefficient (ADC), AD, and radial diffusivity (RD), were calculated for the ACC, CC, and thalamus. In addition to the ROI-based analyses, the cerebrospinal tracts were segmented out from a whole brain tractogram using seed and target ROIs positioned in the motor cortex and pons, respectively (Wright et al. 2017). The segmented left and right corticospinal tracts (CSTs) (L-CST and R-CST, respectively) were then sampled at seven equidistant points and the mean FA value was determined at each point for statistical analysis.

### Statistics

Two-way ANOVAs with diet (standard; HFHS) and stress (control; MS) as factors were run for all baseline behavioral measures using SPSS 25 for MAC. Three-way ANOVAs with diet (standard; HFHS), stress (control; MS), and treatment (sham; surgery) as factors were run for all postsurgical behavioral measures and for the volumetric MRI analyses. Post hoc pairwise comparisons were run when appropriate. Although male and female animals were included, sex was not run as a factor due to insufficient power. A MANOVA was run for the diffusion MRI metrics with diet (standard; HFHS), stress (control; MS), and treatment (sham; surgery) used as dependent variables. For the MRI tractography analyses of the R-CST and L-CST, respectively, we used Mplus 8.3 for Mac (Muthen and Muthen 2012) to understand whether diet (standard; HFHS), stress (control; MS), and treatment (sham; surgery) predicted tractography activity alongside seven measurement points of the R-CST and L-CST. We included auto-regressive effects (i.e., controlled for tractography activity at the previous measurement point of the right and left cerebrospinal tracts) to model change alongside the right and left cerebrospinal tracts. Moreover, we also assessed whether tractography activity alongside the seven measurement points of the R-CST and L-CST predicted our measures of thermal nociceptive responsivity (hot plate at 52 °C and cold plate at 2 °C) as well as our measures of mechanical nociceptive responsivity (von Frey left and right). All estimates of this analysis will be presented as standardized regression weights. All figures are displayed as means ± standard error and statistical significance was considered, *P* < 0.05. The data used in this analysis can be found at DOI 10.17605/OSF.IO/YARQT.

## Results

### Baseline Behavioral Results

Body weight at euthanasia was consistent with the HFHS diet phenotype, whereby animals that consumed the HFHS diet and animals fed the standard diet were significantly different (*P* < 0.01); however, weight was unaltered by the MS exposure early in life or by the plantar incision surgery (*P*s > 0.05). At baseline, the early life manipulations (HFHS and MS) significantly influenced anxiety-like behavior in the EPM and interacted to alter pain sensitivity measured with the hot cold plate and von Frey filaments.

#### EPM—Time in Open Arms

**Figure 2:**
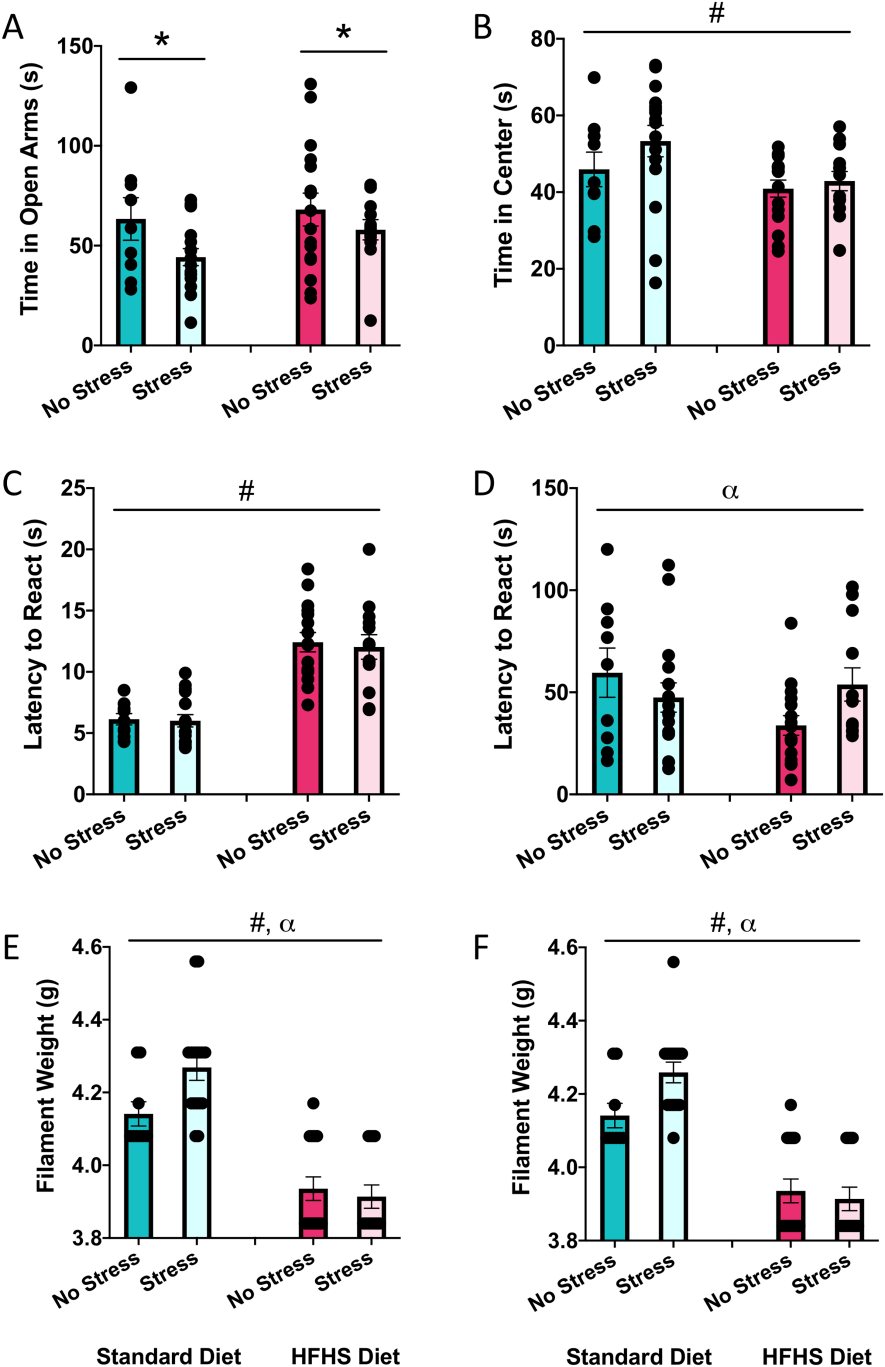
Graphical representation of behavioral results at the baseline timepoint. (*A*) Average time spent in the open arms of the EPM, (*B*) average time spent in the center of the EPM, (*C*) average latency to react to the hot plate, (*D*) average latency to react to the cold plate, (*E*) average filament weight to react to the von Frey on the left paw, and (*F*) average filament weight to react to the von Frey on the right paw. Means, along with individual data points, $\pm$standard error are depicted; * indicates a main effect of stress, # indicates a main effect of diet, and ⍺ indicates a significant diet × stress interaction, *P* < 0.05.

The two-way ANOVA for the time spent in the open arms demonstrated that animals in the MS group had evidence of increased anxiety-like behavior when compared with animals in the control group, as exhibited by a main effect of stress, *F*(1, 52) = 4.07, *P* = 0.05. There were no main effects of diet, *F*(1, 52) = 1.61, *P* = 0.21, or an interaction, *F*(1, 52) = 0.39, *P* = 0.54. See Figure 2*A*.

#### EPM—Time in Center

The two-way ANOVA for time spent in the center demonstrated that animals in the HFHS group spent less time in the center of the maze when compared with animals on the standard diet, *F*(1, 52) = 4.96, *P* = 0.03. There were no main effect of stress, *F*(1, 52) = 1.82, *P* = 0.18, or an interaction, *F*(1, 52) = 0.61, *P* = 0.44. See Figure 2*B*.

#### Hot Plate

The two-way ANOVA for latency to react demonstrated that the animals on the HFHS diet exhibited a significantly increased latency to respond when compared with animals on the standard diet, *F*(1, 53) = 63.42, *P* < 0.01. There were no main effects of stress, *F*(1, 52) = 0.11, *P* = 0.74, or an interaction, *F*(1, 52) = 0.03, *P* = 0.86. See Figure 2*C*.

#### Cold Plate

The two-way ANOVA for latency to respond demonstrated a significant interaction between diet and stress, *F*(1, 52) = 4.31, *P* = 0.04, whereby MS reduced the response time in standard diet animals but increased the latency to respond in HFHS-diet animals. There were no main effects of diet, *F*(1, 53) = 1.57, *P* = 0.22, or stress, *F*(1, 52) = 0.26, *P* = 0.61. See Figure 2*D*.

#### von Frey (Left and Right Paws)

Results from the two-way ANOVAs for filament weight in the von Frey task for the left and right hind paws were nearly identical, exhibiting a main effect of diet (left—*F*(1, 53) = 63.34, *P* < 0.01; right—*F*(1, 52) = 71.01, *P* < 0.01) and a significant interaction between diet and stress (left—*F*(1, 53) = 4.50, *P* = 0.04; right—*F*(1, 52) = 4.56, *P* = 0.04). See Figure 2*E*,*F*. The results indicate that animals fed the HFHS diet are significantly more sensitive in the von Frey task when compared with animals fed with the standard diet. In addition, while MS reduced sensitivity in standard-fed animals, it had no effect in the HFHS-diet animals. There were no main effects of MS for the left or right paw (left—*F*(1, 53) = 2.26, *P* = 0.14; right—*F*(1, 52) = 2.15, *P* = 0.14).

### Postsurgical Behavioral Outcomes

The surgical procedure significantly influenced the pain sensitivity for both paws in the von Frey task and interacted with stress to modify the outcomes in the hot plate. However, surgery did not have an effect on the anxiety or latency to respond in the cold plate. Although MS and the HFHS diet continued to influence the anxiety-like behaviors and pain sensitivity, the outcomes were differentially modified at this second time point.

#### EPM—Time in Open Arms

**Figure 3:**
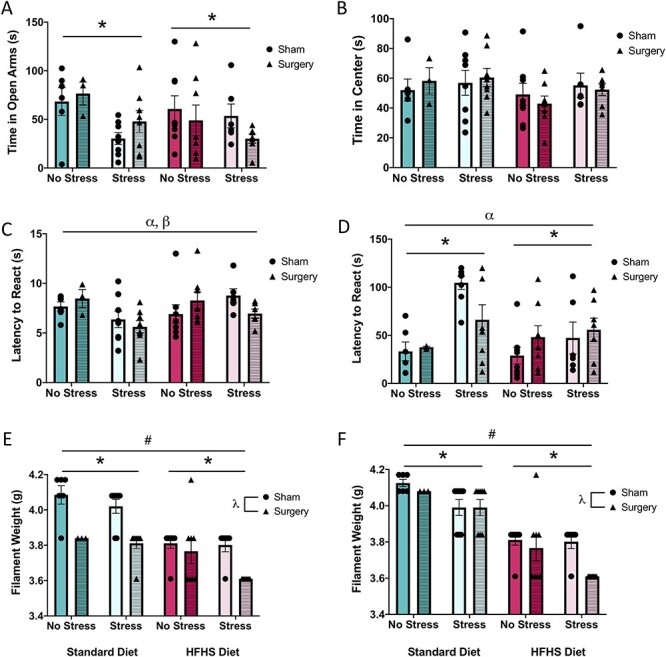
Graphical representation of behavioral results at the postsurgical timepoint. (*A*) Average time spent in the open arms of the EPM, (*B*) average time spent in the center of the EPM, (*C*) average latency to react to the hot plate, (*D*) average latency to react to the cold plate, (*E*) average filament weight to react to the von Frey on the left paw, and (*F*) average filament weight to react to the von Frey on the right paw. Means, along with individual data points, $\pm$standard error are depicted; * indicates a main effect of stress, # indicates a main effect of diet, $\lambda$ indicates a main effect of treatment, ⍺ indicates a significant diet × stress interaction, and $\beta$ indicates a significant stress × treatment interaction, *P* < 0.05.

The three-way ANOVA for time spent in the open arms demonstrated that animals in the MS group were significantly more anxious than animals in the control group, as exhibited by a main effect of stress, *F*(1, 53) = 6.63, *P* = 0.01. There were no main effects of diet, *F*(1, 53) = 0.68, *P* = 0.42, treatment, *F*(1, 53) = 0.07, *P* = 0.80, or any interactions, *P*s > 0.05. See Figure 3*A*.

#### EPM—Time in Center

The three-way ANOVA for time spent in the center demonstrated no main effects, (diet—*F*(1, 53) = 1.78, *P* = 0.19; stress—*F*(1, 53) = 1.18, *P* = 0.29; treatment, *F*(1, 53) = 0.01, *P* = 0.97) or an interaction, *P*s > 0.05. See Figure 3*B*.

#### Hot Plate

The three-way ANOVA for latency to respond demonstrated no main effect (diet—*F*(1, 53) = 1.44, *P* = 0.24; stress—*F*(1, 53) = 2.43, *P* = 0.13; treatment, *F*(1, 53) = 0.03, *P* = 0.87), but it did reveal a significant diet by stress interaction, *F*(1, 53) = 4.11, *P* = 0.05, and a significant stress by treatment interaction, *F*(1, 53) = 4.18, *P* = 0.05. See Figure 3*C*. The diet by stress interaction demonstrates that in standard-fed animals, stress reduced the latency to respond, while in HFHS-diet animals, stress increased the latency to respond. Further, the stress by treatment interaction illustrates that for control animals the surgical procedure increased response latency, whereas the surgical procedure reduced response latency in MS animals. None of the other interactions were significant, *P*s > 0.05.

#### Cold Plate

For latency to respond, the three-way ANOVA revealed a main effect of stress, *F*(1, 53) = 12.14, *P* < 0.01, and a significant diet by stress interaction, *F*(1, 53) = 4.19, *P* = 0.05. See Figure 3*D*. The significant results demonstrate that animals in the MS group had increased response latencies compared with control animals and that, while standard fed animals exposed to MS exhibited increased response latencies, this was not evident in the MS + HFHS-diet animals.

#### von Frey (Left and Right Paws)

As in the baseline measure, the von Frey results for the left and right paws were consistent. The three-way ANOVAs demonstrated a main effect of diet (left—*F*(1, 53) = 35.53, *P* < 0.01; right—*F*(1, 52) = 84.35, *P* < 0.01), stress (left—*F*(1, 53) = 4.07, *P* = 0.05; right—*F*(1, 52) = 9.01, *P* < 0.01), and treatment (left—*F*(1, 53) = 28.78, *P* < 0.01; right—*F*(1, 52) = 4.68, *P* = 0.04). See Figure 3*E*,*F*. The surgical procedure, MS, and the HFHS diet, all significantly reduced the filament weight required to respond. There were no significant interactions for either paw, *P*s > 0.05.

### In vivo MRI Outcomes

#### Volumetric Analyses

**Figure 4:**
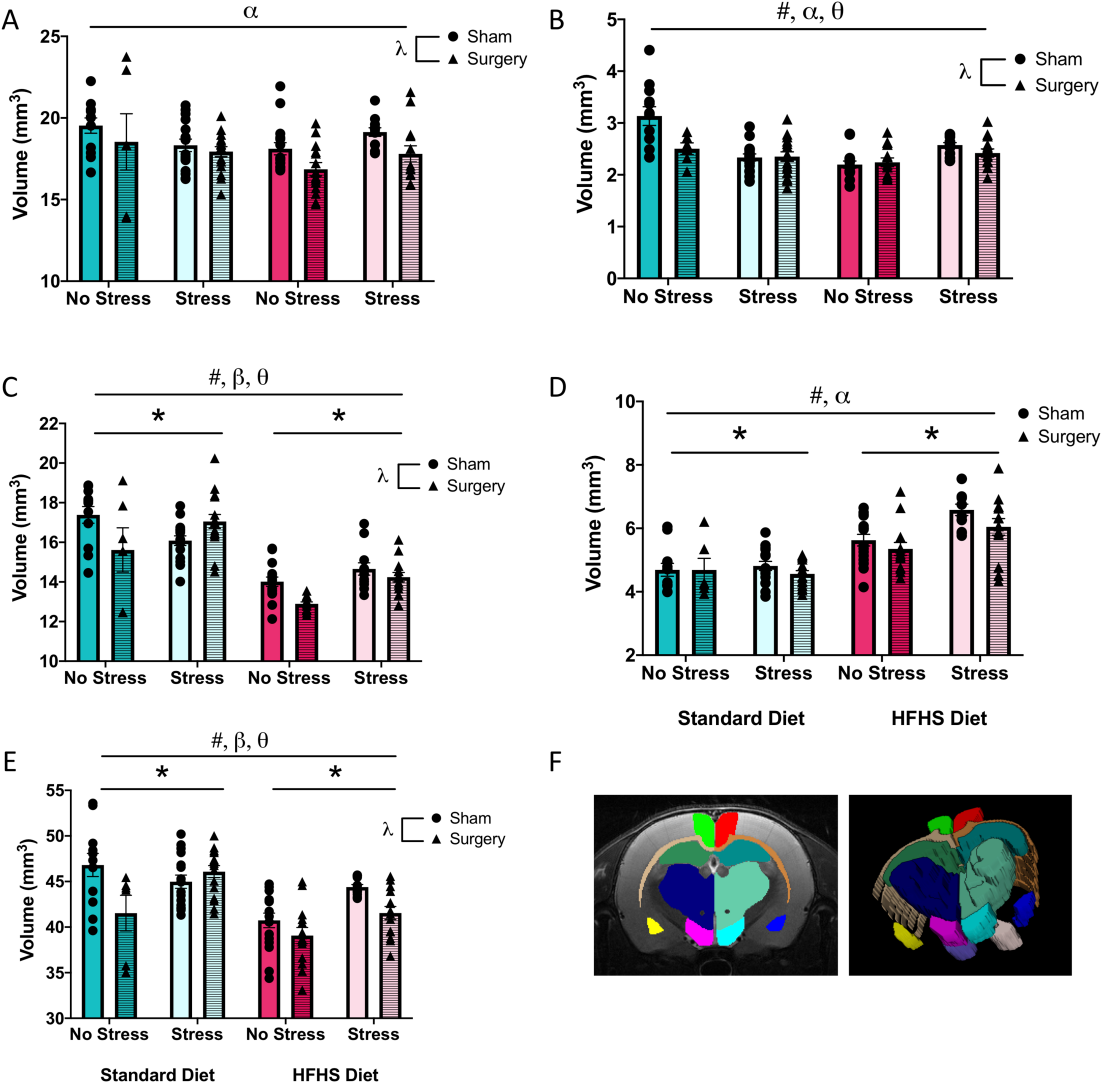
Graphical representation of volumetric measures from MRI analysis. (*A*) Average volume of the ACC, (*B*) average volume of the amygdala, (*C*) average volume of the CC, (*D*) average volume of the NAc, (*E*) average volume of the thalamus, and (*F*) visual representation of regions segmented. Means, along with individual data points, $\pm$standard error are depicted; * indicates a main effect of stress, # indicates a main effect of diet, $\lambda$ indicates a main effect of treatment, ⍺ indicates a significant diet × stress interaction, $\beta$ indicates a significant stress × treatment interaction, and $\theta$ indicates a significant diet × stress × treatment interaction, *P* < 0.05.

Overall, the HFHS diet and the surgical treatment reduced volume in all brain regions (ACC, amygdala, CC, and thalamus) except in the NAc, where animals exhibited an increase in volume. Conversely, early life MS reduced the CC volume but increased the volume in the NAc and the thalamus. Two-way interactions were identified in all brain regions, while three-way interactions between diet, stress, and treatment were observed for the amygdala, CC, and thalamus. See Table 1 for the summary of statistical findings and Figure 4 for an illustrative representation. These interactions suggest that these early life experiences modify the response to a painful surgical procedure, thereby altering the developmental processes and subsequent volume of the critical brain regions.

**Table 1:** Results from the three-way ANOVAs (including F statistic and P value) for the MRI volumetric analysis.

Brain region	Diet F (*P*)	Stress F (*P*)	Treatment F (*P*)	Significant interactions F (*P*)
ACC	2.98 (0.09)	0.01 (0.91)	7.99 (<0.01)	Diet × stress: 7.19 (<0.01)
Amygdala	10.02 (<0.01)	1.98 (0.16)	6.60 (0.01)	Diet × stress: 28.76 (<0.01) 3-Way inter: 9.03 (<0.01)
CC	111.57 (<0.01)	4.67 (0.03)	5.64 (0.02)	Stress × treat: 12.27 (<0.01) 3-Way inter: 4.31 (0.04)
NAc	70.52 (<0.01)	8.17 (<0.01)	3.43 (0.07)	Diet × stress: 8.18 (<0.01)
Thalamus	28.77 (<0.01)	12.05 (<0.01)	11.63 (<0.01)	Stress × treat: 4.18 (0.04) 3-Way inter: 8.83 (<0.01)

#### Diffusion Analyses

**Figure 5:**
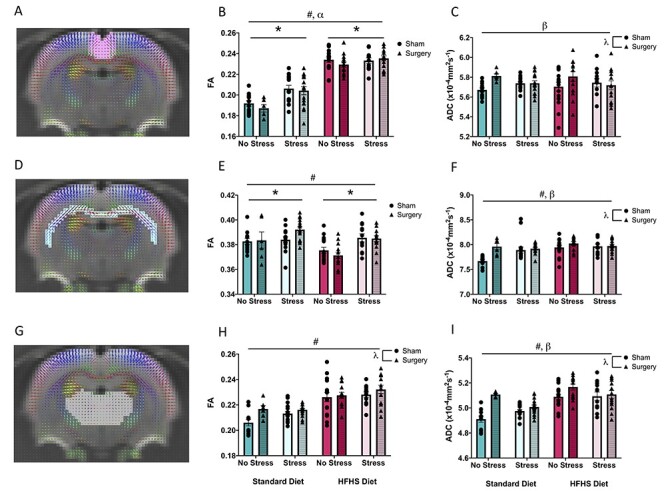
Graphical representation of diffusion tensor measures, FA and ADC from MRI analysis. (*A*) Visual representation of diffusion in the ACC, (*B*) average FA in the ACC, (*C*) average ADC in the ACC, (*D*) visual representation of diffusion in the CC, (*E*) average FA in the CC, (*F*) average ADC in the CC, (*G*) visual representation of diffusion in the thalamus, (*H*) average FA in the thalamus, and (*I*) Average ADC in the thalamus. Means, along with individual data points, $\pm$standard error are depicted; * indicates a main effect of stress, # indicates a main effect of diet, $\lambda$ indicates a main effect of treatment, ⍺ indicates a significant diet × stress interaction, and $\beta$ indicates a significant stress × treatment interaction, *P* < 0.05.

Mean diffusion tensor metrics, including FA, ADC, RD, and AD, were calculated for the ACC, CC, and thalamus. See Table 2 for the summary of statistical results and Figure 5 for an illustrative representation of the FA and ADC results. Overall, we found that consumption of the HFHS modified nearly all diffusion metrics (with the exception of ADC in the ACC), whereas early life MS only influenced FA (in the ACC and the CC) and RD (in the CC). The acutely painful surgical procedure had a substantial effect on the numerous brain connectivity measures across all three brain regions. Moreover, two- and three-way interactions suggested that the early life manipulations interacted with the surgical procedure to modify the functional connectivity within the developing brain.

**Table 2:** Results from the MANOVA (including F statistic, P value, and *partial ETA squared*) for the MRI diffusion analysis.

Brain region	Diffusion metric	Diet F (*P*), partial ETA-squared	Stress F (*P*), partial ETA-squared	Treatment F (*P*), partial ETA-squared	Significant interactions F (*P*), partial ETA-squared
ACC	FA	242.30 (<0.01) *0.712*	15.83 (<0.01) *0.139*	1.01 (0.32) *0.010*	Stress × diet: 8.13 (<0.01) *0.077*
	ADC	0.02 (0.90) *0.000*	0.25 (0.62) *0.003*	3.95 (0.05) *0.039*	Stress × treat: 5.94 (0.02) *0.057*
	RD	19.38 (<0.01) *0.165*	2.59 (0.11) *0.026*	5.21 (0.03) *0.050*	Stress × treat: 7.15 (<0.01) *0.068*
	AD	49.07 (<0.01) *0.334*	1.65 (0.20) *0.017*	1.33 (0.25) *0.013*	None
*CC*	FA	9.85 (<0.01) *0.091*	17.66 (<0.01) *0.153*	0.28 (0.60) *0.003*	None
	ADC	12.88 (<0.01) *0.116*	1.34 (0.25) *0.013*	9.56 (<0.01) *0.089*	Stress × treat: 6.62 (0.01) *0.063*
	RD	35.42 (<0.01) *0.265*	3.94 (0.05) *0.039*	1.96 (0.17) *0.020*	Stress × diet: 6.09 (0.02) *0.059* Stress × treat: 8.18 (<0.01) *0.077*
	AD	30.11 (<0.01) *0.235*	0.59 (0.45) *0.006*	5.32 (0.02) *0.051*	Stress × diet: 5.40 (0.02) *0.052* Stress × treat: 9.13 (<0.01) *0.085*
Thalamus	FA	64.07 (<0.01) *0.395*	2.89 (0.09) *0.029*	6.15 (0.02) *0.059*	None
	ADC	43.37 (<0.01) *0.307*	1.67 (0.20) *0.017*	21.43 (<0.01) *0.179*	Stress × treat: 10.78 (<0.01) *0.099*
	RD	8.71 (<0.01) *0.082*	2.80 (0.10) *0.028*	9.74 (<0.01) *0.090*	Stress × treat: 6.45 (0.01) *0.062*
	AD	122.06 (<0.01) *0.555*	0.09 (0.77) *0.001*	35.72 (<0.01) *0.267*	Stress × treat: 13.55 (<0.01) *0.121* Diet × treat: 6.18 (0.02) *0.059* Stress × diet × treat: 4.32 (0.04) *0.042*

#### Tractography Analyses

**Figure 6:**
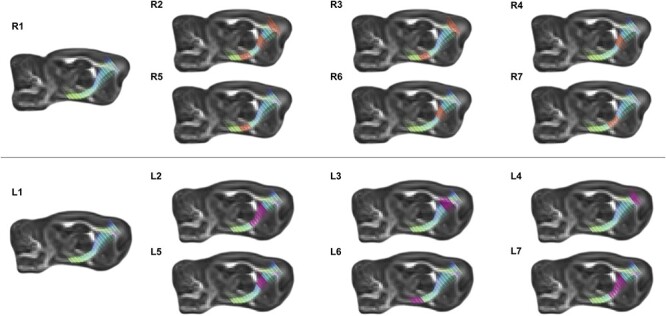
Illustrative representation of FA diffusion along the right (upper panel) and left (lower panel) corticospinal tract using advanced MRI tractography. R1, representative image of the R-CST. For R2–R7, regions of significance are highlighted in orange. R2, regions of the R-CST where FA diffusivity is positively correlated with consumption of the HFHS diet; R3, regions of the R-CST where FA diffusivity is positively correlated with MS; R4, regions of the R-CST where FA diffusivity is positively correlated with surgical treatment; R5, regions of R-CST where FA diffusivity is negatively correlated with thermal nociception on the hot plate; R6, regions of R-CST where FA diffusivity is negatively correlated with thermal nociception on the cold plate; and R7, regions of R-CST where FA diffusivity is negatively correlated with mechanical nociception in the right paw von Frey task. L1, representative image of the L-CST. For L2–L7, regions of significance are highlighted in magenta. L2, regions of the L-CST where FA diffusivity is positively correlated with consumption of the HFHS diet; L3, regions of the L-CST where FA diffusivity is negatively correlated with consumption of the HFHS diet; L4, regions of the L-CST where FA diffusivity is positively correlated with MS; L5, regions of L-CST where FA diffusivity is positively correlated with surgical treatment; L6, regions of L-CST where FA diffusivity is negatively correlated with mechanical nociception in the left paw von Frey task; and L7, regions of L-CST where FA diffusivity is negatively correlated with mechanical nociception in the right paw von Freytask.

The CSTs are the primary voluntary motor pathways connecting the brain to the body and are critical for nociception. With respect to the R-CST, we found that a HFHS diet was positively associated with tractography activity at measurement point 1 (β = 0.667, standard error [SE] = 0.087, *P* < 0.001), point 2 (β = 0.390, SE = 0.134, *P* < 0.001), point 4 (β = 0.181, SE = 0.110, *P* = 0.040), and point 6 (β = 0.324, SE = 0.119, *P* = 0.020) after controlling for previous tractography activity; all other associations were nonsignificant. See Figure 6—R2. We moreover found that MS stress was positively associated with tractography activity at the measurement point 1 (β = 0.211, SE = 0.097, *P* = 0.020), point 2 (β = 0.295, SE = 0.137, *P* = 0.020), and point 6 (β = 0.224, SE = 0.109, *P* = 0.030) after controlling for previous tractography activity; all other associations were nonsignificant. See Figure 6—R3. Finally, we found that surgery was positively associated with tractography activity at measurement point 4 (β = 0.004, SE = 0.002, *P* < 0.001) after controlling for previous tractography activity; all other associations were nonsignificant. See Figure 6—R4. With respect to the effect of tractography activity alongside the seven measurement points of the R-CST, we found that tractography activity at measurement point 1 (β = 0.561, SE = 0.217, *P* = 0.010) was positively associated with the thermal nociceptive responsivity to the hot plate at 52 °C, whereas tractography activity at measurement point 6 (β = −0.599, SE = 0.217, *P* = 0.010) was negatively associated with the thermal nociceptive responsivity hot plate at 52 °C; all other associations were nonsignificant. See Figure 6—R5. Moreover, we found that tractography activity at measurement point 4 (β = −0.270, SE = 0.204, *P* = 0.030) was negatively associated with the thermal nociceptive responsivity cold plate at 2 °C; all other associations were nonsignificant. See Figure 6—R6. Next, we found that tractography activity at measurement point 1 (β = −0.358, SE = 0.196, *P* = 0.040) was negatively associated with the mechanical nociceptive responsivity for von Frey left; all other associations were nonsignificant. Finally, we found that tractography activity at measurement point 1 (β = −0.371, SE = 0.190, *P* = 0.030) and measurement point 5 (β = −0.349, SE = 0.187, *P* = 0.010) was negatively associated with the mechanical nociceptive responsivity for von Frey right; all other associations were nonsignificant. See Figure 6—R7.

With respect to the L-CST, we found that a HFHS diet was positively associated with tractography activity at measurement point 1 (β = 0.676, SE = 0.083, *P* < 0.001), measurement point 4 (β = 0.401, SE = 0.088, *P* < 0.001), measurement point 5 (β = 0.257, SE = 0.116, *P* = 0.020), see Figure 6—L2, whereas it was negatively associated with tractography activity at measurement point 3 (β = −0.147, SE = 0.075, *P* = 0.020) after controlling for previous tractography activity; all other associations were nonsignificant. See Figure 6—L3. We moreover found that MS stress was positively associated with tractography activity at measurement point 2 (β = 0.291, SE = 0.141, *P* = 0.020) after controlling for previous tractography activity; all other associations were nonsignificant. See Figure 6—L4. Finally, we found that surgery was positively associated with tractography activity at measurement point 4 (β = 0.004, SE = 0.002, *P* = 0.010) after controlling for previous tractography activity; all other associations were nonsignificant. See Figure 6—L5. With respect to the effect of tractography activity alongside seven measurement points of the L-CST, we found that tractography activity at measurement point 1 (β = 0.300, SE = 0.166, *P* = 0.040) was positively associated with the thermal nociceptive responsivity on the hot plate at 52 °C; all other associations were nonsignificant. Moreover, we found no significant associations between the tractography activity and the thermal nociceptive responsivity on the cold plate at 2 °C. Next, we found that tractography activity at measurement point 3 (β = 0.247, SE = 0.119, *P* = 0.030) was positively associated with the mechanical nociceptive responsivity for von Frey left, whereas tractography activity at measurement point 7 (β = −0.284, SE = 0.122, *P* = 0.010) was negatively associated with the mechanical nociceptive responsivity for von Frey left; all other associations were nonsignificant. See Figure 6—L6. Finally, we found that tractography activity at measurement point 1 (β = −0.230, SE = 0.138, *P* = 0.040), measurement point 4 (β = −0.208, SE = 0.124, *P* = 0.030), and measurement point 5 (β = −0.446, SE = 0.188, *P* < 0.001) was negatively associated with the mechanical nociceptive responsivity for von Frey right, see Figure 6—L7; all other associations were nonsignificant.

## Discussion

Owing to increased neuroplasticity and a variety of programming mechanisms, early life factors have remarkable potential to influence outcomes in adolescence and beyond (Welberg and Seckl 2001). Given that poor diet and ACEs increase inflammation and the release of stress hormones, prime microglia, and the HPA axis to overrespond to later stressors (Kalinichev et al. 2002; Aisa et al. 2007; McGowan et al. 2009; Hains et al. 2010; Calder et al. 2011; Diz-Chaves et al. 2013; Buckman et al. 2014; Baumeister et al. 2016; Edlow et al. 2019), we hypothesized that poor diet and ACEs would modify the pain response in adolescence and alter recovery from an acutely painful stimulus. To test this, we investigated a variety of behavioral as well as structural and functional neurobiological outcomes with known roles in nociception. Our baseline behavioral results support our hypotheses. First, we found that a HFHS diet altered both thermal and mechanical nociceptive responsivity, as seen on the hot cold plate and von Frey tasks, respectively. This is consistent with the high-fat diet literature (Loredo-Pérez et al. 2016; Tramullas et al. 2016; Song et al. 2017; Song et al. 2018) and is likely occurring in response to increased systemic inflammation, as high-fat diets have been shown to increase the levels of proinflammatory cytokines, reactive oxygen species, and glial markers (Ohsawa et al. 2004; Shapiro et al. 2009; White et al. 2009; Igosheva et al. 2010; Pistell et al. 2010; André et al. 2017). Nociceptive thresholds may be lowered as inflammation activates normally “silent” unmyelinated C nociceptors (Schmidt et al. 1995). While most nociceptive tests trigger Aδ fibers, the more numerous silent C fibers are susceptible to inflammatory pain and become sensitized by inflammatory mediators, thus reducing pain thresholds (Schmidt et al. 1995; Le Bars et al. 2001). Second, results from baseline testing with respect to the MS paradigm demonstrated that MS increased anxiety on the EPM and altered nociceptive responsivity on the cold plate and von Frey measures. It is not surprising that MS altered these measures, as ACEs are strongly correlated with pain, anxiety, and other forms of psychological distress (Hovens et al. 2010; Kessler et al. 2010; Tietjen et al. 2010; Green et al. 2011; Scott et al. 2011; Beal et al. 2020; Beveridge et al. 2020). While early stress can program neurobiological systems to cope with stress in the long term, studies show that if there is a mismatch in circumstances, this programming is maladaptive and leads to pain, anxiety, and other psychological and behavioral challenges (Welberg and Seckl 2001; Korosi et al. 2012; Burke et al. 2013; Banqueri et al. 2017; Vilela et al. 2017). Consistent with our findings, anxiety has been associated with pain problems in adolescence (Kessler et al. 2007; Shelby et al. 2013; Soltani et al. 2019; Battaglia et al. 2020). Further, by altering levels of glucocorticoid receptors, stress has been demonstrated to increase proinflammatory cytokines and reactive microglia, which influences pain thresholds (McGowan et al. 2009; Diz-Chaves et al. 2013; Baumeister et al. 2016), while stress-induced increases in microglia activation have also been associated with heightened anxiety (Gracia-Rubio et al. 2016; Mizoguchi et al. 2019). Therefore, our results support much of the current literature that has found associations between ACEs and the increased risk for anxiety- and pain-related disorders in adolescence (Davis et al. 2005; Sachs-Ericsson et al. 2007; Shelby et al. 2013; Noel et al. 2016; Nelson et al. 2017; Sachs-Ericsson et al. 2017; Battaglia et al. 2020). Results in the literature, however, do vary based on methodology; factors such as type of early stress, nociceptive tests conducted, age, and sex all contribute to the outcomes. For instance, different forms of early stress, such as brief separations of early handling (<15 min) actually decrease nociceptive sensitivity (Stephan et al. 2002). This may be due to early exposure to small amounts of stress preparing the pups for later stressors. Longer periods of separation, such as in maternal separation or deprivation (1–24 h), tend to support our findings with increased sensitivity (see Chen and Jackson 2016 for review).

Following demonstration that these two early life factors altered baseline emotional and pain responsivity, we investigated the same outcomes following administration of an acutely painful stimulus in adolescence. This was done to determine if these early experiences could modify the neurobiological nociceptive response, as this may aid in our understanding of factors that contribute to the development of chronic pain. As expected, the HFHS diet and ACE altered recovery from the minor surgical procedure. The increased anxiety and mechanical nociceptive sensitivity induced by MS continued, providing supportive evidence for the long-lasting effects of ACEs. MS also interacted with the surgical treatment to alter thermal sensitivity, whereby maternally stressed animals that also underwent the surgical procedure exhibited exacerbated pain outcomes. As MS and other ACEs alter HPA axis function and induce basal proinflammatory states (Kalinichev et al. 2002; Aisa et al. 2007; Diz-Chaves et al. 2013), the immune system is likely primed and overresponds to minimal stressors (McGowan et al. 2009; Diz-Chaves et al. 2013; Baumeister et al. 2016), such as a minor surgical procedure. Similarly, the chronic low-grade inflammation often induced by HFHS diets have been shown to prime microglia to overrespond to later stressors (Hains et al. 2010; Calder et al. 2011; Buckman et al. 2014; Edlow et al. 2019). Under normal conditions, microglia activation is a necessary immune response that protects the central nervous system, phagocytosing damaged cells, and releasing proinflammatory cytokines (Smith et al. 2012; Baufeld et al. 2016; Peng et al. 2016; Hickman et al. 2018). However, systemic inflammation induced by poor diet can lead to chronic activation of this microglial response, which leaves the system vulnerable and unable to respond correctly when triggered by acute events (Hickman et al. 2018). It is therefore not surprising that the most prominent and exaggerated pain response was identified in animals that experienced both MS and HFHS diet consumption prior to the acutely painful stimulus. Additionally, the exacerbated von Frey effects were seen in both the left (paw which underwent surgical procedure) and right (no surgery) paws, suggesting a systemic nociceptive response to the surgical stressor rather than a change in local sensitization. These additive effects are important to note, as this mirrors results from clinical populations, whereby the cumulation of ACEs significantly increase the risk for chronic pain problems (You et al. 2019).

Not only did these early life experiences have lasting effects on nociception-related behavioral measures, they also produced changes in the brain structure and function. Using in vivo MRI to examine the ACC, amygdala, CC, NAc, and thalamus, our results support current literature indicating that early life stress and diet alter the volume and diffusivity of numerous brain regions (Cohen et al. 2006; Spinelli et al. 2009; Duque et al. 2012; Baker et al. 2013; Hanson et al. 2013; Howell et al. 2013; Lou et al. 2014; Saleh et al. 2017; Croll et al. 2018). First, we found that the HFHS diet altered volume in the CC, NAc, amygdala, and thalamus, all of which play a significant role in reward processing and emotional regulation, which are both affected by early life stress and consumption of a HFHS diet (Volkow et al. 2011; Ward 2013; Silverman et al. 2015; Anderson et al. 2017; Novick et al. 2018). For instance, consumption of hyper-palatable foods increases dopamine stimulation and release (Volkow et al. 2011). In an effort to adapt and compensate for this continuous release of dopamine, many regions of the brain downregulate the expression of dopamine receptors, which ultimately leads to a blunted response to future rewards (Volkow et al. 2011). In addition, HFHS diets have also been associated with numerous factors known to influence cerebral volume, such as decreased neuronal resiliency, reduced neuroplasticity, and changes in brain-derived neurotrophic factor expression and synaptic plasticity (Molteni et al. 2002; Pistell et al. 2010; Boitard et al. 2015; Reichelt et al. 2019).

Second, we found that MS modified volumetric measures in the CC, NAc, ACC, and thalamus. Similar to HFHS diets, early life stress has also been associated with emotional dysregulation and increased risk for substance abuse disorders (Dube et al. 2003; Phillips et al. 2005; Andersen and Teicher 2009). Stress dysregulates the HPA and corticotropin-releasing factor (CRF) systems, altering levels of glucocorticoids throughout the reward circuit, and thus affecting downstream neurotransmission (Lupien et al. 2009; Barik et al. 2013). Further, changes in CC volumes are consistently found in clinical studies of children exposed to childhood maltreatment, another significant ACE (Daniels et al. 2013). Although the exact mechanisms driving the relationship between stress and modified brain volume have yet to be fully elucidated, it is thought that dysregulation of the HPA axis plays a substantial role (Lupien et al. 2009). The dysregulation of glucocorticoid secretion and HPA axis activity decreases spine density, reduces dendritic length and branching, and suppresses neurogenesis (McKittrick et al. 2000; Radley et al. 2004; Radley et al. 2006; Holmes and Wellman 2009; Lupien et al. 2009), all of which contribute to region-specific changes in the brain volume.

Third, and most remarkably, despite the fact that there were only 10 days between the minor surgical procedure and the *in vivo MRI*, animals in the surgery groups exhibited a reduction in ACC, amygdala, CC, and thalamic volume, as well as increased diffusion in the ACC, CC, and thalamus. Furthermore, the numerous significant interactions we identified between the treatment, HFHS, and MS highlight that the surgery-induced changes in the neurobiological structure and function are mediated by the animal’s early experiences. Although clinical studies have been able to use changes in the white matter connectivity, and specifically FA, to predict the chronification of pain (Mansour et al. 2013), they have not been able to identify the underlying predisposing factors that drive these changes in white matter diffusivity in their human cohorts. Our results suggest that early life experiences may prime the brain to respond abnormally to what would otherwise be considered a relatively mild insult. Consistent with this, previous research has found that stress and corticosteroids suppress mitosis of glial cells (Gould et al. 1992; Jauregui-Huerta et al. 2010) and therefore affect myelination and white matter tract development, leading to the modification of FA in the CC and thalamic regions (Lim et al. 2020). Moreover, given that HFHS diets promote systemic inflammation and thus increased microglia activation (Calder et al. 2011; Buckman et al. 2014; Cope et al. 2018) and that microglia are important for remodeling white matter and dendritic spine density, increased activation results in altered connectivity and synaptic pruning (Cope et al. 2018) and consequently changes in diffusivity.

Finally, as thermal and mechanical nociception primarily involve the ventral posterior thalamus and relay through the CST (Boivie et al. 1989; Bowsher 1996), we also examined diffusivity of the CST in response to our experimental manipulations. The CSTs begin in the cerebral cortex (the primary motor and somatosensory cortices) and descend through the brainstem where they give rise to the pyramidal tracts of the spinal cord. Traumatic brain injuries often induce spontaneous pain (hyperalgesia, allodynia, etc.) which has been linked to changes in FA along the CSTs (Goto et al. 2008; Jang and Seo 2020). It is therefore not surprising that we found FA differences in the CSTs of our animals, which could be directly attributed to changes in nociception, the surgical procedure, and the early life manipulations. Our results demonstrating that changes in different regions of the CSTs are associated with distinctive pain responses are consistent with prior literature. For example, a study conducted by Liu et al. found that a particular subset of corticospinal neurons that originate in the somatosensory cortices and innervate the spinal cord via the CST are specifically responsible for mechanically induced allodynia but not other aspects of pain sensitivity, such as cold allodynia (Liu et al. 2018). In addition, following nerve injury, there was an increase in the number of these neurons that were activated when mechanically stimulated with von Frey filaments (Liu et al. 2018). This may explain our increased FA for area 4 of both the R-CST and L-CST in response to the surgical procedure, as the changes in diffusion may reflect region-specific hyperactivity of corticospinal neurons. However, to our knowledge, this is the first study to demonstrate that early life experiences (both HFHS diet and MS) can induce changes in FA along the CSTs that are associated with differences in nociceptive responsivity. Interestingly, prior studies have demonstrated that prenatal experiences that increase neuroinflammation, such as gestational diabetes (Ornoy 2005), pregnancy-induced hypertension (Kronenberg et al. 2006), and intrauterine inflammation (O'shea et al. 2009) can disrupt the microstructural development of the CSTs. Although not directly examined here, neuroinflammation may be the key factor underlying the negative effects of early adversity on neurobiological function and should be investigated in greater detail.

## Conclusion

In summary, our data suggest that both a HFHS diet and the ACE (MS) modified the pain response in adolescence and recovery from an acutely painful stimulus. Further, MRI analyses demonstrated that not only did exposure to the HFHS diet and MS modify the brain structure and function, but these adverse early experiences also altered the neurobiological response to the minor surgical procedure. Therefore, we conclude that early life factors, such as diet and ACEs, have the potential to impact long-term emotional and nociceptive outcomes by altering the functional connectivity within the brain. However, further studies should be conducted to comprehensively characterize the neuroinflammatory mechanisms that could be underlying the changes in nociception and brain maturation. In addition, although our study included both males and females, it may not have been sufficiently powered to fully interrogate the sex differences in the trauma–pain relationship, and this requires additional attention. Moreover, the pups in our study were maintained on the diet to which they were born in an attempt to best represent a clinical setting, whereby children are likely to consume the same meals as their parents. However, this design prevents us from being able to distinguish any additive effects that may have arisen from consumption of the diet in the pre + postnatal environments. Future studies may seek to tease these intergenerational effects apart with a criss-cross design study, whereby the diet of pups is switched at birth. Nevertheless, this study highlights the significant role that early experiences play in nociception-related behavioral and neurobiological outcomes, and given that these adverse early experiences are modifiable (improved diet, maternal support to prevent ACEs, etc.), understanding their role in pain processing provides us with a target for early intervention and may help reduce the burden of chronic pain in adolescence and across the lifespan.

## Notes

The authors acknowledge the facilities and scientific and technical assistance of the National Imaging Facility (NIF), a National Collaborative Research Infrastructure Strategy (NCRIS) capability at Monash Biomedical Imaging (MBI), a Technology Research Platform at Monash University. *Conflict of Interest:* The authors have no competing financial interests with respect to the work described here.
